# Impact of Exposure to Environmental Enrichment on the Anxiety-Like Behavior of Ovariectomized Mice

**Published:** 2020-01

**Authors:** Alfredo Briones-Aranda, Manuela Castellanos-Pérez, Victor Manuel Vega- Villa, Ofir Picazo

**Affiliations:** 1 Pharmacology Laboratory, Faculty of Human Medicine, Autonomous University of Chiapas, Tuxtla Gutiérrez, Chiapas, México.; 2 Sección de Estudios de Posgrado e Investigación, Escuela Superior de Medicina, Instituto Politécnico Nacional, México City, México.

**Keywords:** *Anxiety-Like Behavior*, *Ovariectomized Mice*, *Physical Enrichment*, *Social Enrichment*

## Abstract

**Objective:** The aim of this study was to explore the influence of short-term (2-week) exposure to social (SE) and/or physical enrichment (PE) on the anxiety-like behavior of ovariectomized (OVX) NIH Swiss mice.

**Method** **:** One week after surgery, each OVX mouse was housed under one of 4 social conditions: (1) isolated, (2) accompanied by an intact female, (3) accompanied by an intact male, or (4) in a community of 10 OVX individuals. The animals in each of these environments were divided into 2 subgroups, consisting of the presence and absence of PE. Following a 2-week exposure to the respective conditions, each OVX mouse was subjected to either the light/dark exploration test (LDT) or the elevated plus maze (EPM) to examine anxiety-like behavior.

**Results: **The LDT and EPM showed very similar patterns. Compared to an impoverished environment, PE elicited a significant anxiolytic effect for OVX mice housed alone or in companion of an intact female (F [1, 54] = 16.11, P = 0.001). By contrast, mice living in community but without PE displayed anxiogenic-like behavior, perhaps due to crowding, compared to the animals living in isolation (F [1, 36] = 5.64, P = 0.023).

**Conclusion: **This study emphasized the importance of taking housing conditions into account during the screening of new anxiolytic agents and the critical role of OVX in the regulation of anxiety.

An ovariectomy (OVX) has been shown by different tests to produce an increase in anxiety-like behavior in rodents (1, 2), which can be attenuated by hormonal replacement (3, 4). Therefore, removal of the ovaries obviates cyclic variations and it is used to evaluate anxiety regulation in females (5, 6).

Treatment of ovariectomized rats (7, 8) and mice (9) with ovarian hormones (estradiol and progesterone) is known to have an anxiolytic-like effect, as illustrated by a variety of behavioral tests. A similar anxiolytic-like behavior is described for rodents during proestrus versus diestrus (7). Such hormonal anxiety regulation is more evident in rodents experiencing drastic changes in ovarian activity due to aging or removal of the ovaries. 

In line with the above, other studies on adult female rodents indicated that the administration of estradiol and progesterone is linked to anxiolytic-like effects (10, 11, 12). However, a small number of studies found an anxiogenic-like effect after giving progesterone (13) or estradiol (14) to adult female rodents. 

On the other hand, anxiety-like behavior can also be regulated by environmental enrichment (EE), consisting of physical enrichment (PE) and/or social enrichment (SE). Enrichment of physical surroundings is generally accomplished by providing the home cage with toys or objects that favor voluntary exercise and exploratory behavior (15). Enrichment of the social ambience involves housing 2 or more animals in the same cage to stimulate social interaction (16, 17). Both conditions have proven to attenuate the stress generated in rodents when living in isolation (18, 19 and 20). Although EE seems to be associated with an anxiolytic-like effect for both male (21, 22) and female (23, 24) rodents, several factors are able to influence such an effect, especially the rodent species or strain (25, 26), gender (27, 28), and the serum levels of ovarian hormones (23).

While most studies utilizing the OVX mouse model and behavioral tests have evidenced a pro-cognitive effect of EE (29), few have independently examined the conditions of SE or PE in relation to the regulation of anxiety in OVX rodents (16, 20), and none, to our knowledge, have determined the outcome of different combinations of SE and PE on the anxiety-like behavior of OVX mice. 

Hence, the aim of this study was to evaluate the effect of a short-term (2-week) exposure to social (SE) and/or physical enrichment (PE) on the anxiety-like behavior of OVX mice. The effect of various combinations of SE and PE was herein analyzed by means of 2 well-validated techniques to assess anxiety, the light/dark exploration test (LDT), and the elevated plus maze (EPM).

## Materials and Methods

***Animals***

NIH Swiss mice, purchased from the Harlan animal house of the National Autonomous University of Mexico (Mexico City), were used to generate breeding stock and experimental animals in the local vivarium of the Autonomous University of Chiapas. The animals were freed of all viral, bacterial, and parasitic pathogens listed in the recommendations by the Federation of European Laboratory Animal Science Association (30). The mice, taken from distinct litters, were randomly assigned to various groups. Female and male mice (5-6 weeks old, 25-30 g) were housed in acrylic static isolation cages (Soluciones MG, Mexico City, Mexico) containing pine tree chips as bedding (Grigamex, Mexico City, Mexico). All mice were submitted to a 12:12 hour inverted light cycle (lights on at 7 PM). Mice had unlimited access to standard rodent chow (LabDiet 5001, St. Louis, MO, USA) and purified water (provided in bottles). All procedures were approved by the institutional bioethics committee and were performed in accordance with the guidelines of the Mexican norm for animal protection (NOM-062-ZOO-1999).

***Ovariectomy***

The OVX procedure was performed on 200 mice interperitoneally administered 25 mg/kg sodium pentobarbital (Pisa Pharmaceutical, Hidalgo and Mexico) to produce general anesthesia. After shaving and disinfecting the surgical site, a 0.5-cm ventral midline incision was made with a scalpel. Subsequently, the connective tissue was separated to cut through the peritoneal wall. The ovaries were located and removed bilaterally; then, the peritoneum and the muscle layers were stitched with an absorbable suture (Ethicon chromic sutures-4/0, Johnson & Johnson Medical Devices, USA) and the skin with a nonabsorbable nylon suture (Ethicon mononylon sutures-4/0, Johnson & Johnson Medical Devices, USA) that spontaneously detached at 2-3 days post surgery.

***Experimental Design***

Following one week of recovery from OVX, all animals were assigned to one of 8 groups, which were designed for the purpose of testing the effect of different forms and combinations of EE. There were 4 social environments, consisting of isolation (an OVX mouse housed alone in a cage) and 3 distinct types of SE. In the latter cases, an OVX mouse was housed with an intact male, with an intact female, or in a community of 10 OVX individuals. Each of the 4 social conditions was subdivided into 2 physical environments, with and without PE (Table 1).

However, these 8 groups were not all used in the same experiment. There were 2 experiments presently conducted, each with a distinct set of groups to examine the effect of EE on the anxiety-like behavior of OVX mice. 

For community housing, each of the mice was marked with a number (using a non-toxic permanent marker) in the dorsal region and then put in a large cage (60 cm x 40 cm x 22 cm). For the community group exposed to PE, 10 objects for exploration/exercise were scattered throughout the cage. The objects, consisting of plastic tubes (15 cm long x 4 cm in diameter), rectangular plastic blocks of diverse colors and textures (5 cm x 3 cm x 1 cm), rattles and glass marbles (2 cm in diameter) were randomly moved every other day. The groups with 1 or 2 individuals were put in a small cage (33 cm x 14 cm x 14 cm), which for the condition of PE contained only 1 object. In all cages, the object or objects were carefully cleaned and randomly moved every other day. 

The first experiment compared the consequences of living under 2 general conditions: in isolation (one OVX mouse per cage) or in the company of another mouse (an intact male or female). Accordingly, there were 3 social environments, each subdivided into 2 groups (with or without PE). Hence, 6 independent groups were formed for each behavioral test (LDT and EPM) for a total of 12 groups and 120 animals (10 per group). 

The second experiment analyzed the results of living in isolation versus in community (in each case with or without PE). Thus, 4 independent groups existed for each behavioral test (LDT and EPM) for a total of 8 groups and 80 animals (10 per group). 

***Behavioral Tests***

After 2 weeks of the respective housing conditions, the behavioral evaluations were performed in the 2 anxiety tests. Each animal was subjected to only one of these tests (Figure1). All experiments were done in a room with red light that was isolated from noise. The sessions were recorded and the video was subsequently reviewed (manually) by a trained observer who was blind to the particular group tested. Two groups of mice were subjected to the tests each day from 9 to 11 AM. The researcher only entered the room to place or remove the animals for the tests and carefully clean the test equipment with a solution of 10% ethyl alcohol.

**The Light/Dark Exploration Test**

The LDT is conducted in an acrylic cage (44 x 21 x 21 cm) divided into a relatively small dark compartment constituting 1/3 of the total cage size, and a larger and highly illuminated compartment (560 lx light intensity) occupying 2/3 of the cage. The dark and light areas are separated by a wall with a small opening (13 x 15 cm). For the test, each mouse is introduced (only once) into the light area and the number of transitions through the opening is registered during 10 minutes. An anxiolytic-like effect is considered with a larger number of transitions. The present procedure, chosen as a result of our previous studies (31, 32), is similar to a method published in 1980 (33). 

***The Elevated Plus Maze***

The EPM is based on the natural tendency of mice to actively explore a new environment, countered by a fear of being in an open area. The apparatus consists of a central platform (5 x 5 cm) connecting 2 open arms (25 x 5 cm) with 2 equal-sized enclosed arms (25 x 5 x 15 cm) that form a cross. The maze is elevated to a height of 50 cm above the ground and illuminated by a red light. The evaluation lasts for 5 minutes, beginning when a mouse is placed in the center of the apparatus. A longer time spent on the open arms reflects an anxiolytic-like effect and a longer time on the enclosed arms an anxiogenic-like effect. The total number of crossings from the open to closed arms (or vice versa) is taken as an indicator of ambulatory activity (34).

***Ambulatory Activity Test***

To examine possible alterations in motor activity, a spontaneous ambulatory activity test was performed after the LDT. For this test, the animal is put in an acrylic cage (60 x 40 x 40 cm) with a checkerboard pattern (20 x 20 cm) on the floor, and the total number of squares crossed by the mouse is manually counted for 5 minutes.

***Data Analysis***

All data were analyzed by 2-way ANOVA on SigmaStat v 3.5 (Systat Software, Inc., CA and USA). PE was assigned as factor A and SE as factor B. In both experiments, factor A was defined as the presence or absence of PE and factor B as one of the housing conditions investigated herein (individual, accompanied by an intact male or female, or in a community of 10 OVX mice; see table 1). Post-hoc comparisons were conducted with the Tukey test. A p value <0.05 was considered as statistically significant.

## Results

***Experiment 1***

For OVX mice living in isolation or with an intact female, PE increased the number of transitions in the LDT (Figure 2, upper panel). According to the statistical analysis, anxiety-like behavior was dependent on PE (F [1, 54] = 16.11, P = 0.001), SE (F [2, 54] = 9.06, P = 0.001), and the interaction of both physical and social factors (F [2, 54] = 3.80, P = 0.029). Whether with or without PE, post-hoc comparisons demonstrated a greater number of transitions made by OVX mice accompanied by an intact female than those living in isolation. For the OVX mice with an intact male companion, there was no significant difference in this parameter when comparing the 2 physical environments (impoverished and enriched).

The results of the EPM (Figure 2, lower panel) were similar to those of the LDT for OVX mice housed in isolation or with an intact female companion, with or without PE. The statistical analysis revealed that the time spent on the open arms was dependent on PE (F [1, 54] = 18.74, P = 0.001) and SE (F [2, 54] = 9. 81, P = 0.001), but not on the interaction of both factors (F [2, 54] = 0.22, P = 0.803). These findings are identical to those shown in the LTD, according to the multiple comparison analysis. 

***Experiment 2***

**Figure 3:**
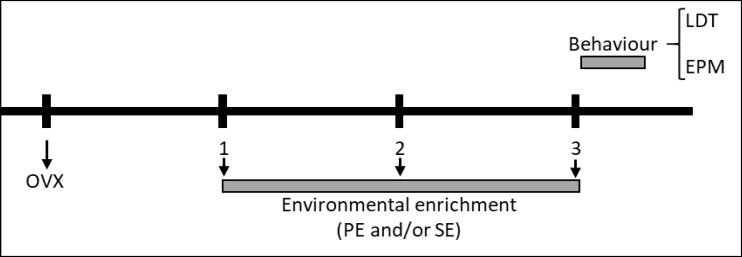
Time Course of the Experiment (The Numbers Refer to Weeks). A Week after the Ovariectomy (OVX), the Different Groups of Animals Was Exposed to Physical Enrichment (PE) and/or Social Enrichment (SE) for 2 Weeks. Upon Completion of this Time, Anxiolytic-Like Behavior Was Evaluated by the Light/Dark Exploration Test (LDT) or the Elevated Plus Maze (EPM).

For OVX mice living in isolation or in community, the presence or absence of PE influenced the number of transitions in the LDT (Figure 3, upper panel) and the time on open arms in the EPM (lower panel). The results of the statistical analysis revealed that anxiety-like behavior found on the LDT was dependent on PE (F[1, 36] = 43.29, P = 0.001) and SE (F[1, 36] = 5.64, P = 0.023), but not on the interaction of both factors (F[1, 36] = 0.67, P = 0.418). Paired comparisons for the groups subjected to impoverished conditions indicated a lower number of transitions for the animals in community housing versus isolation. Independently of the SE condition (community vs. isolation), PE increased the number of transitions. Regarding spontaneous activity, no statistical difference was detected between groups (Table 2).

The pattern of mouse behavior on the LDT and the EPM was identical (considering isolation versus community living and the presence versus absence of PE). A significant influence on anxiety-like behavior was exerted by PE (F [1, 36] = 56.74, P = 0.001) and SE (F [1, 36] = 4.42, P = 0.043), but not by the interaction of both factors (F [1, 36] = 1.53, P = 0.223). A shorter time was spent on the open arms by the mice housed in community versus in isolation, independently of the factor of PE (compared to a smaller number of transitions on the LDT for the mice living in community versus isolation only under impoverished but not enriched conditions). Apart from this exception, the multiple comparison analysis between groups tested on the EPM demonstrated anxiolytic-like behavior similar to that observed in the LDT. Regarding ambulatory activity, no statistical difference existed between the ambulatory activity test (number of squares crossed) and the EPM (total arm entries) (Table 2).

For OVX mice living under different housing conditions, spontaneous ambulatory activity was measured by counting the number of squares crossed or total arm entries in the EPM. 

Two-way analysis of variance revealed nonsignificance of motor activity in all cases. The results of the 2-way ANOVA showed that for number of squares crossed, PE (F [1,72] = 0.81, P = 0.77), SE (F [3,72] = 0.104, 

P =0.95), AxB interaction (F [3,72] = 0.133, P =0.94); for the EPM, PE (F [1,72] =0.85, P = 0.35), SE (F[3,72] = 1.60, P =0.19), AxB interaction (F [3,72] = 0.082, P = 0.96).

**Figure:**
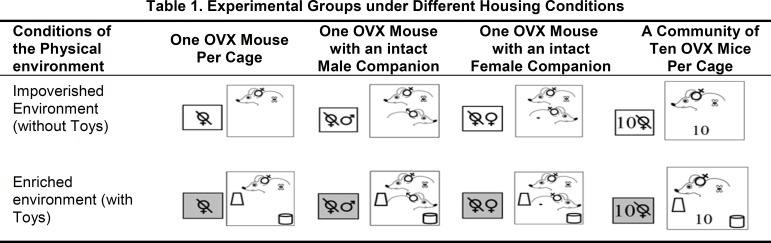


**Figure:**
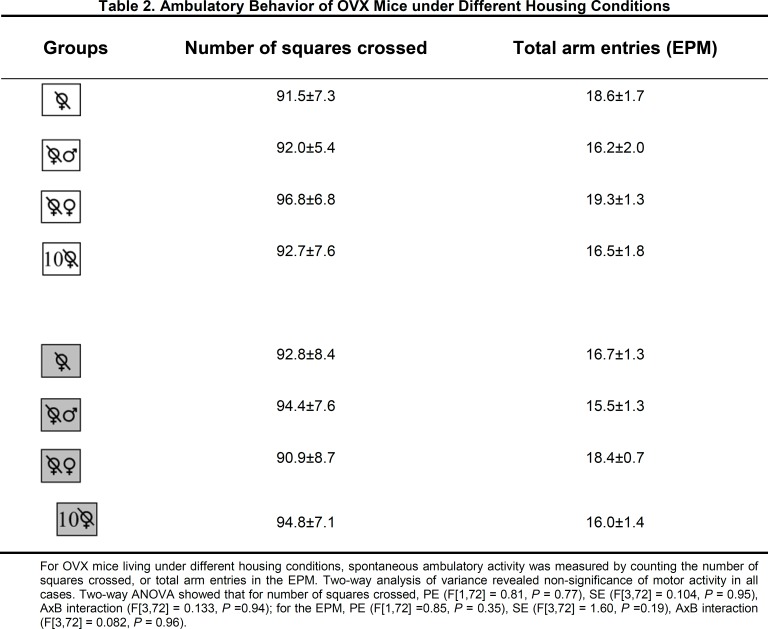


**Figure 4:**
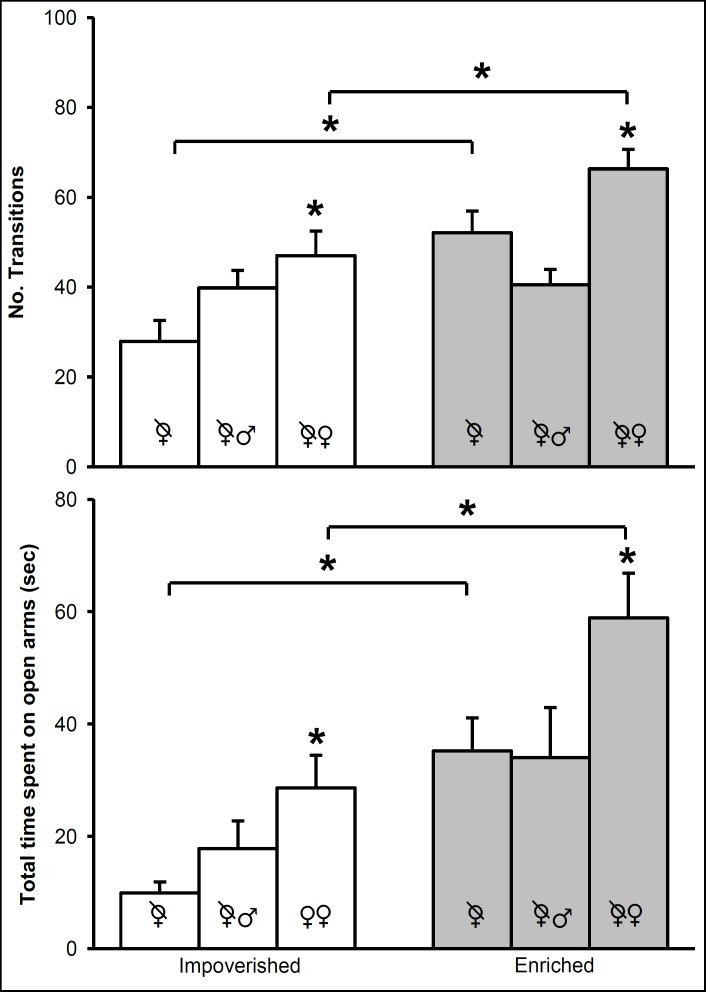
OVX Mice Housed in an Impoverished (Empty Bars) or Enriched Environment (Gray Bars). The upper Panel Depicts the Number of Transitions during 10 Minutes in the LDT. The Lower Panel Portrays the Time Spent by Animals on the Open Arms of the EPM.

Tukey Test, *P < 0.05 *vs* the Corresponding Control Group. Φ = an OVX Mouse, Φ σ = an OVX Mouse + a Male Mouse, Φ ϙ = an OVX Mouse + an Intact Female Mouse.

**Figure 5:**
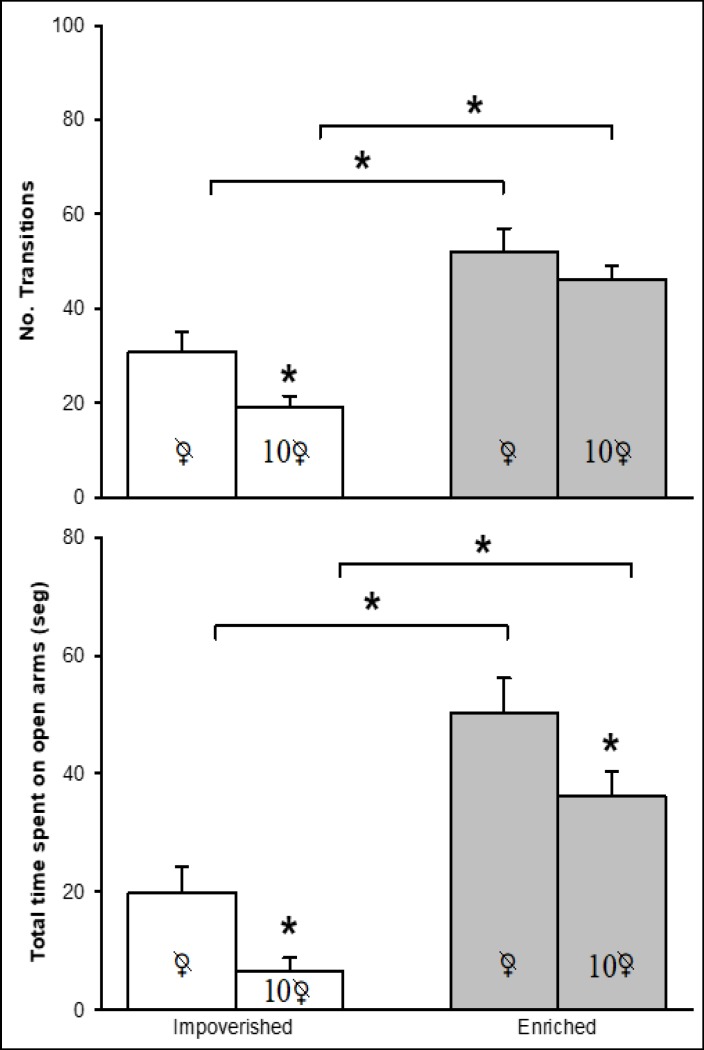
OVX Mice Housed in an Impoverished (Empty Bars) or Enriched Environment (Gray Bars). The upper Panel Depicts the Number of Transitions during 10 Minutes in the LDT. The Lower Panel Portrays the Time Spent by Animals on the Open Arms of the EPM.

Tukey Test, *P<0.05 vs the Corresponding Control Group. Φ = an OVX Mouse, 10 Φ = 10 OVX Mice Housed Together.

## Discussion

Both behavioral tests showed a substantially higher level of anxiety for isolated mice in an impoverished versus enriched environment. Reports in the literature on isolated mice with and without PE have described a similar anxiogenic-like effect for the impoverished environment (35, 36), though none of these studies included isolated OVX mice. 

The removal of ovaries is a known risk factor for the development of anxiety symptoms in rats (6). The current contribution points to the importance of taking housing conditions into account when evaluating the influence of PE.

In the absence of PE, an anxiogenic-like behavior was exhibited by isolated OVX mice, but not by OVX mice accompanied by a gonadal-intact female partner. 

Likewise, it has been documented that pair-housed OVX prairie voles maintain a low level of anxiety compared to isolated animals (37), and that after stressing events male rats manifest less anxiety if living in pairs rather than in isolation (16).

According to the aforementioned reports, SE is significant as long as the companions have similar characteristics (eg, gender, endocrine profile, and prior experiences). However, anxiogenic-like behavior was herein displayed by OVX mice living in community (10 per cage) in the absence of PE, which may have been related to the smaller space available per mouse under such conditions (even though the overall space was larger). Indeed, an increase in housing density of mice is a well-known stress factor (38) linked to anxiogenic-like behavior (39).

There have been descriptions in the literature of mice housed under conditions similar to those used herein, but with distinct results (20, 40). This discrepancy might have been due to various methodological factors, including animal strain, cage size, coexistence time, sex, hypoestrogenism, etc. (25, 41). For example, the sensitivity to stress may be greater for hypogonadal animals than for their intact counterparts (42), potentiating the negative consequences of overcrowding and thus provoking high levels of anxiety. Sexual behavior in male mice (attacks and stimulating genital sniffs and mounts) occurs in the presence of an intact or OVX female (43). After pairing an OVX mouse with an intact male under impoverished conditions, the behavioral tests seem to indicate stress for the female. Contrarily, an anxiolytic-like effect was detected when an OVX mouse was housed with an intact mouse of the same sex, also under impoverished conditions. Hence, the sex of the partner appears to influence the anxiety levels of OVX mice in paired living. In the future, it would be recommendable to consider these findings in the design of protocols to screen new drugs with possible anxiolytic properties.

Except for the OVX mice with a male partner, the beneficial impact of PE was clearly demonstrated in this study, in line with previous reports of a PE-induced reduction in behavioral and physiological responses to anxiogenic stimuli (40, 44). For OVX mice accompanied by a male partner, on the other hand, PE apparently could not compensate for the anxiety-like effect of the male presence, perhaps caused by the aggressive behavior of the males to the OVX females, especially the attacks, genital sniffs, and mounts (43). In this sense, prior investigation has associated an overproduction of corticosterone with a greater susceptibility of OVX rodents to stressful events (42), which is in agreement with data on OVX rats practicing voluntary running (as a factor of PE). Although such activity does not alter anxiety-like behavior, it increases corticosterone levels (15). Interestingly, these can be blocked by estrogens (15). Further research is needed to explore the capacity of estrogens to elicit an anxiolytic-like effect in OVX mice living with a male partner and with PE.

The evaluation of experiments was herein limited to the observation of anxiolytic- and anxiogenic-like behavior. Since biochemical analysis was not performed, there was no interpretation of possible mechanisms of the findings. In the future, it is necessary to employ a paradigm of OVX mice housed in isolation and in distinct social conditions in the presence and absence of PE, and attention should be paid on measuring the activity of some neurotransmitters (eg, GABA, glutamate and serotonin) and the expression of certain receptors (eg, GABA A and 5-HT1A).

## Limitation

The evaluation of experiments was herein limited to the observation of anxiolytic- and anxiogenic-like behavior. Since biochemical analysis was not performed, there was no interpretation of possible mechanisms of the findings.

## Conclusion

The results of this study emphasized the importance of OVX in the regulation of anxiety and perhaps in responsiveness to stress. Additionally, different social environments affected the anxiety-like behavior of OVX mice in the presence and absence of PE. Thus, it is necessary to conduct studies using a biochemical approach to provide insights into the mechanisms involved in the regulation of anxiety when OVX animals are housed under distinct social and physical contexts.
